# Serum 25-Hydroxyvitamin D Status and Vitamin D Supplements Use Are Not Associated with Low Back Pain in the Large UK Biobank Cohort

**DOI:** 10.3390/nu16060806

**Published:** 2024-03-12

**Authors:** Sha Sha, Li-Ju Chen, Hermann Brenner, Ben Schöttker

**Affiliations:** 1 Division of Clinical Epidemiology and Aging Research, German Cancer Research Center (DKFZ), Im Neuenheimer Feld 581, 69120 Heidelberg, Germany; sha.sha@dkfz-heidelberg.de (S.S.); li-ju.chen@dkfz-heidelberg.de (L.-J.C.); h.brenner@dkfz-heidelberg.de (H.B.); 2 Faculty of Medicine, University of Heidelberg, Im Neuenheimer Feld 672, 69120 Heidelberg, Germany; 3 Division of Preventive Oncology, National Center for Tumor Diseases (NCT), Im Neuenheimer Feld 581, 69120 Heidelberg, Germany

**Keywords:** low back pain, 25-hydroxyvitamin D, vitamin D supplement use, real-world evidence

## Abstract

Longitudinal studies assessing the association of vitamin D deficiency, defined by serum 25-hydroxyvitamin D levels < 30 nmol/L, and vitamin D supplement (VDS) use with low back pain (LBP) are sparse. This investigation assessed the cross-sectional and longitudinal association of vitamin D status and VDS use with LBP among 135,934 participants from the UK Biobank cohort. Approximately 21.6% of the participants had vitamin D deficiency, while only 4% regularly took VDS. In the month before study enrollment, 3.8% of the population reported experiencing LBP. An additional 3.3% of the population were diagnosed with LBP by their general practitioners for the first time during a median follow-up of 8.5 years. Vitamin D deficiency and VDS use were cross-sectionally associated with LBP in age- and sex-adjusted models, but these associations were not evident in comprehensively adjusted models. In longitudinal analyses, both vitamin D deficiency and VDS use were not associated with LBP in any model after correction for multiple testing. In conclusion, not unexpectedly due to the fact that LBP is multifactorial, our findings provide no evidence for the role of the vitamin D status in the etiology of LBP.

## 1. Introduction

The widespread prevalence of low vitamin D status poses a significant public health concern across many countries [1,2,3]. Vitamin D is known to offer a wide range of health benefits [4]. Its integral role in maintaining musculoskeletal health, including facilitating calcium absorption, bone mineralization, and supporting muscle function, is widely acknowledged [5]. For example, it was observed in a primary care clinic in Minneapolis, USA, that 93% of patients with musculoskeletal disorders had insufficient 25-hydroxyvitamin D (25[OH]D) levels (≤50 nmol/L) [6], the most commonly used and accurate measure of vitamin D concentration in the body. Apart from musculoskeletal health, vitamin D also holds potential influence on pain modulation due to its anti-inflammatory role [7,8]. Studies have shown that individuals with various chronic pain conditions often exhibit lower serum levels of 25(OH)D [4,9].

As the most prevalent musculoskeletal disorder, low back pain (LBP) has emerged as the leading cause of years lived with disability worldwide [10]. Recent estimates indicate that approximately 7.5% of the world’s population experienced LBP in 2017, a number that is on the rise [10]. The incidence of LBP has been increasing across all age groups since 1990, with a more pronounced increase among the middle-aged population [11]. In developed countries, up to 90% of individuals may experience LBP at some point in their lives, leading to substantial medical and socioeconomic costs [10,12,13].

LBP is a multifactorial condition, which can be caused by a sedentary lifestyle, psychosocial issues, injuries, comorbidities, occupational reasons, and genetic predisposition or a mix of these [14,15]. It is of interest whether a sufficient vitamin D status could play a role in the prevention of such a multifactorial condition at all.

A meta-analysis of 28 observational studies observed a cross-sectional association of vitamin D deficiency with LBP [16]. However, most included studies had limitations, such as small sample sizes and inadequate adjustment for confounders [16]. While there was no separate meta-analysis with longitudinal studies conducted, a recently published prospective study failed to corroborate the association [17], leading to the presumption that the cross-sectional association between 25(OH)D levels and LBP is not causal.

In addition to observational studies, a Mendelian randomization study demonstrated an association between genetically predicted 25(OH)D levels and a reduced risk of LBP in the European population [18]. In contrast, a meta-analysis of eight small clinical trials involving vitamin D supplementation as an intervention did not show efficacy in treating LBP [19].

Notably, to our knowledge, there is also a lack of real-world evidence from large datasets examining the longitudinal association of vitamin D supplement use with the onset of LBP. Therefore, this study used data from the large, population-based United Kingdom (UK) Biobank cohort to comprehensively investigate the associations of 25(OH)D status and the use of vitamin D supplements with LBP, both cross-sectionally and longitudinally.

## 2. Materials and Methods

### 2.1. Study Population

This investigation used data sourced from the UK Biobank, a prospective cohort comprising over half a million individuals from the UK. The participants, aged between 40–69 years, were recruited from 2006 to 2010 across 22 assessment centers situated in England, Scotland, and Wales [20]. The UK Biobank has amassed an extensive collection of biomedical information through processes including electronically endorsed consent forms, touch-screen questionnaires, brief interviews, diverse physical and functional evaluations, and analyses of blood, urine, and saliva specimens [20,21]. Continuous follow-up of health-related outcomes is facilitated through linkages to numerous medical records, including UK National Health Service (NHS) data, primary care data, cancer screening data, and disease-specific registers [22,23].

### 2.2. Vitamin D Status

Serum 25(OH)D status was categorized based on the thresholds proposed by the Institute of Medicine in the United States: vitamin D deficiency was defined as having a 25(OH)D concentration of less than 30 nmol/L, vitamin D insufficiency as 25(OH)D between 30 and less than 50 nmol/L, and sufficient vitamin D status as 25(OH)D of 50 nmol/L or higher [24]. Quantification of 25(OH)D concentration in serum was carried out by chemiluminescence immunoassay on the DiaSorin Liaison XL platform manufactured by Diasorin S.p.A., Saluggia, Vercelli, Piedmont, Italy [24]. The results were further verified through the Randox International Quality Assessment Scheme (RIQAS) Immunoassay Speciality I scheme, attesting to the robustness of the quality control measures implemented [24,25].

### 2.3. Vitamin D Supplements Use

We obtained data regarding vitamin D and multivitamin supplement use from the questionnaire administered by the UK Biobank at baseline visit. The questionnaire included the query: “Do you regularly take any of the following (You can select more than one answer)?” (Data Field 6155). The response options of “Vitamin D” and “Multivitamins with or without minerals” were collected for analyses. Approximately 82% of vitamin D users received vitamin D supplements over the counter (OTC), and 18% by prescription.

### 2.4. Low Back Pain

We collected LBP date and diagnosis information from primary care records up to the date of the last data extraction from the NHS primary care dataset (September 2017) [23]. We identified the diagnosis of LBP using the 10th revision of the International Statistical Classification of Diseases (ICD-10) code M54.5 before the baseline visit for cross-sectional analyses, and during the follow-up period for longitudinal analyses. In cross-sectional analyses, we further checked the self-reported back pain from the questionnaire assessment of the UK Biobank via the query “Pain type(s) experienced in last month”, with one of the options being “Back pain” (Data-Field 6159). By combining diagnosed LBP from primary care records with self-reported information about back pain experienced in the last month, an exposure variable of physician-diagnosed LBP with acute symptoms at baseline was created for cross-sectional analysis.

### 2.5. Covariates

In our previous analyses of the UK Biobank cohort [26], a large number of variables statistically significantly associated with vitamin D deficiency (n = 48) or vitamin D supplement use (n = 49) were identified. These encompassed a broad spectrum, including not only sociodemographic factors, lifestyle factors, body mass index (BMI), biomarkers, diseases and health-related factors, but also vitamin D-specific considerations such as the geographic latitude of the assessment center and seasonality (i.e., the calendar month of attending assessment centers and blood draw) [26]. Adherence to a healthy lifestyle and the season of assessment are also relevant for LBP and are crucial aspects to adjust for [15,27,28,29]. The covariates included in the final full models were selected from these determinants of vitamin D deficiency or vitamin D supplement use, along with two additional factors specific to LBP: a history of other musculoskeletal diseases before baseline enrollment (ICD-10: M00-M53, M55-M99) and injuries to the abdomen, lower back, lumbar spine, and pelvis prior to baseline (ICD-10: S30-S39) from primary care data [15]. The statistical method for the covariate selection is outlined in Section 2.7.2. The final list of covariates adjusted for in the models is shown in Table S1, Supplemental Materials, and the baseline distribution of the covariates is shown in Table S2, Supplemental Materials.

### 2.6. In- and Exclusion Criteria

**Figure 1:**
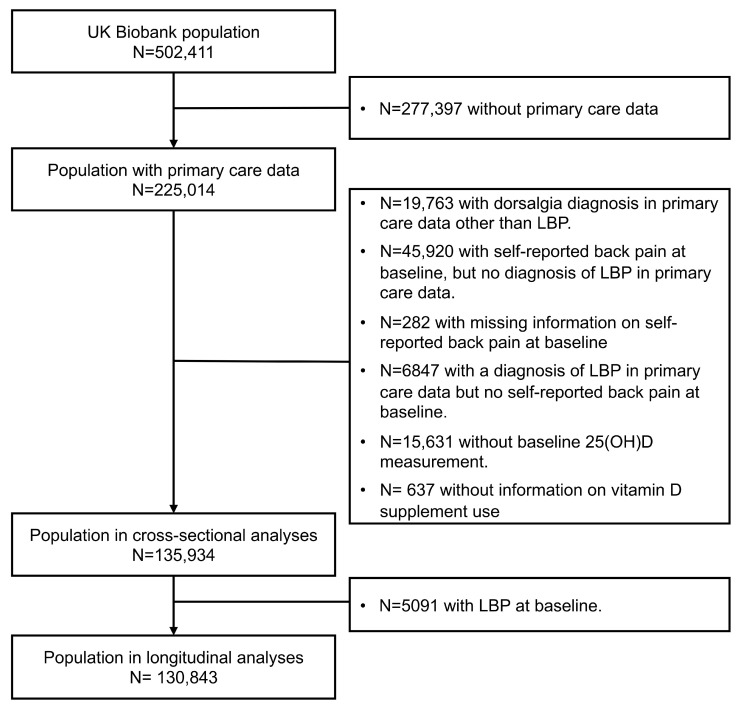
Flow-chart of the study population. Abbreviations: 25(OH)D, 25-hydroxyvitamin D; LBP, low back pain.

To ascertain LBP diagnoses, linkage of the UK Biobank cohort to the NHS primary care data was required. While 98% of the UK population is registered with the NHS, at the time of analysis, the primary care data had not been linked to the IT system supplier EMIS practice [22,23,30]. Among the total study population of n = 502,411 UK Biobank participants, linkage with NHS primary care data was feasible for n = 225,014 study participants (Figure 1). Furthermore, it was necessary to exclude subjects with potentially undiagnosed LBP at baseline. In the baseline questionnaire, subjects were asked about symptoms of back pain in the last month. We decided to compare individuals with physician-diagnosed LBP and acute symptoms at baseline with subjects without any back pain in a cross-sectional analysis. To establish this population, we applied the following exclusion criteria (Figure 1):

1. Individuals with a history of dorsalgia diagnoses other than LBP in the primary care data (diagnosed using the ICD-10: M54.0–54.4, 54.6–54.9) before the baseline assessment (n = 19,763);
2. Self-reported back pain in the last month in the questionnaire (n = 45,920) or missing information about this question (n = 282) unless LBP was diagnosed in the primary care data;
3. History of LBP diagnosis in the primary care data but no current symptoms reported in the question about LBP in the last month (n = 6847).

Additionally, individuals lacking serum 25(OH)D measurements (n = 15,631) or with incomplete information regarding self-reported vitamin D/multivitamin supplements use (n = 637) were excluded. This resulted in a final population of n = 135,934 for the cross-sectional analyses (Figure 1). For the longitudinal analysis, we further excluded n = 5091 individuals with a history of LBP diagnosis and reported back pain in the month prior baseline, leaving a population of n = 130,843.

### 2.7. Statistical Analyses

#### 2.7.1. General Remarks

Statistical analyses in the study were carried out using Statistical Analysis System (SAS) software (version 9.4, SAS Institute, Inc., Cary, NC, USA). Two-sided statistical tests were employed, with a predetermined *p*-value < 0.003125 indicating statistical significance due to correction for multiple testing for 16 tests by the Bonferroni method [31]. The 16 tests are for the four exposures (i.e., vitamin D deficiency, vitamin D insufficiency, vitamin D supplements use and multivitamin use) tested for an association with LBP in both cross-sectional and longitudinal analysis in two models: age- and sex-adjusted model as well as the full model (4 × 2 × 2 = 16 tests).

The handling of missing values involved multiple imputation, using the Markov chain Monte Carlo approach, resulting in the creation of five imputed datasets [32,33]. Analysis of results from imputed datasets was carried out using the SAS procedure ‘PROC MIANALYZE’. In the context of longitudinal analyses, we used Schoenfeld residuals to assess the proportional hazards assumption, revealing no evidence of violations of this assumption.

#### 2.7.2. Covariates Selection

The set of potentially relevant covariates described in Section 2.5 was reduced by a stepwise backward elimination with a stay criterion of *p* < 0.05 using LBP at baseline as the dependent variable in a logistic regression model. Age and sex were forced into the model regardless of statistical significance. Furthermore, variation inflation factors (VIFs) were used to examine the presence of multicollinearity among the variables incorporated in the final models [34].

#### 2.7.3. Association between Vitamin D Status and LBP

We applied logistic regression and Cox proportional hazards regression to assess the cross-sectional and longitudinal association of 25(OH)D status with LBP, respectively. In both cross-sectional and longitudinal analyses, two distinct models were applied. The first model was adjusted for age and sex. The full model comprised the variables obtained by the covariate selection described in Section 2.7.2 (see Table S1, Supplemental Materials, for final list of all covariates).

Furthermore, we assessed the dose–response patterns of 25(OH)D concentrations in association with LBP using the same models, employing restricted cubic splines (RCS) characterized by five knots positioned at the 5th, 25th, 50th, 75th, and 95th percentiles of the actual value of serum 25(OH)D. A 25(OH)D level of 75 nmol/L was used as a point of reference [35,36].

#### 2.7.4. The Association between Vitamin D Supplement Use and LBP

Similar to the analysis of 25(OH)D levels, we also used logistic regression and Cox proportional hazards regression to investigate the cross-sectional and longitudinal association of vitamin D supplement use with LBP, respectively.

#### 2.7.5. Subgroup Analyses

All analyses were further stratified based on age (<65/≥65 years old), sex (female/male), with/without a history of depression, and with/without a history of other musculoskeletal diseases.

## 3. Results

### 3.1. Baseline Overview of the Study Population

Table 1 presents an overview of the baseline characteristics of the study population. The cross-sectional analyses in the study included n = 135,934 participants with a median age of 58 years (interquartile range [IQR]: 50–63). A slightly higher proportion of females (54%) was included compared to males. Approximately 65.4% of the studied population were overweight or obese. Individuals who had never smoked (56.6%) marginally outnumbered those who had smoked. Furthermore, a substantial proportion of participants reported experiencing hypertension (25.8%) and having had depression in their lifetime (10.8%). Less frequently reported comorbidities included diabetes (4.6%) and coronary heart disease (4.3%). The median chronic diseases per individual was 1 (IQR: 0–3). Over half of the participants (53.5%) had been diagnosed with musculoskeletal diseases by general practitioners (GPs) in their lifetime, but only 2% had a lifetime history of an injury to the abdomen, lower back, lumbar spine, or pelvis.

Overall, 3.8% of the population had a GP diagnosis of LBP prior to baseline and reported suffering from LBP in the month before the study enrollment. Regarding vitamin D status, the median concentration of 25(OH)D was 46.3 (IQR: 32.0–61.9) nmol/L, and a significant portion of participants were identified as either vitamin D deficient (21.6%) or insufficient (34.5%). Regular use of vitamin D supplements was reported by only 4% of participants, though a further 19.7% reported regular use of multivitamin (±mineral) preparations.

Excluding the participants with LBP at baseline for the longitudinal analysis did not significantly alter the distribution of baseline characteristics. During the follow-up period, 3.3% of participants received their first LBP diagnosis.

### 3.2. Covariates Associated with LBP at Baseline

Two pools of variables were used to screen for factors statistically significantly associated with LBP at baseline and to be used for the full models. The first pool consisted of n = 48 factors statistically significantly associated with vitamin D deficiency and the second pool included n = 49 factors statistically significantly associated with vitamin D supplement use. The n = 2 factors, specifically relevant for LBP (history of musculoskeletal diseases and injuries), were part of both variable pools. A total of n = 27 factors were selected by backwards selection from the pool of variables for vitamin D deficiency because they were statistically significantly associated with LBP. With respect to vitamin D supplement use, n = 30 variables were selected from the pool of potential covariates because they showed a statistically significant association with LBP. These two sets of selected variables were subsequently used as the covariates for the full models for vitamin D status and vitamin D supplement use (Table S1, Supplemental Materials). Table S3 (Supplemental Materials) shows the associations of covariates selected from both variable pools with LBP at baseline. The median VIF of these factors in this model was 1.7, spanning from 1.0 to 6.1, which raises no concerns regarding multicollinearity as no VIF was >10 [34].

### 3.3. Association of 25(OH)D Status with LBP

Table 2 presents the cross-sectional and longitudinal associations of vitamin D deficiency and insufficiency (compared to vitamin D sufficiency) with LBP. Vitamin D insufficiency was not associated with LBP in any of the analyses. In the age- and sex-adjusted model, vitamin D deficiency was cross-sectionally associated with acute LBP (odds ratio [OR], 95% confidence interval [CI]: 1.13, 1.05–1.22). However, this association was markedly weakened in the full model, which was adjusted for 27 covariates including BMI and diseases, resulting in an OR that was greatly attenuated and a CI that contained the null effect value, suggesting no significant association.

Throughout a median follow-up period of 8.5 years (IQR: 7.8–9.3 years; maximum: 10.8 years), 4288 individuals received their first physician-diagnosed LBP. Contrary to the findings from the cross-sectional analysis, no longitudinal association was observed between vitamin D deficiency and LBP in the age- and sex-adjusted model. The relationship was even inverse in the fully adjusted model, which is not a biologically plausible direction because this would imply a decreased risk of LBP among individuals with vitamin D deficiency. As expected, this was a finding by chance because of multiple testing. After correcting the *p*-value for multiple testing, this inverse association was not statistically significant (*p* > 0.003125).

In subgroup analyses by age, sex, history of depression and musculoskeletal disease, the associations of vitamin D deficiency with LBP were comparable to the findings from the total cohort (Table S4, Supplemental Materials).

### 3.4. Dose–Response Association of 25(OH)D Concentration with LBP

**Figure 2:**
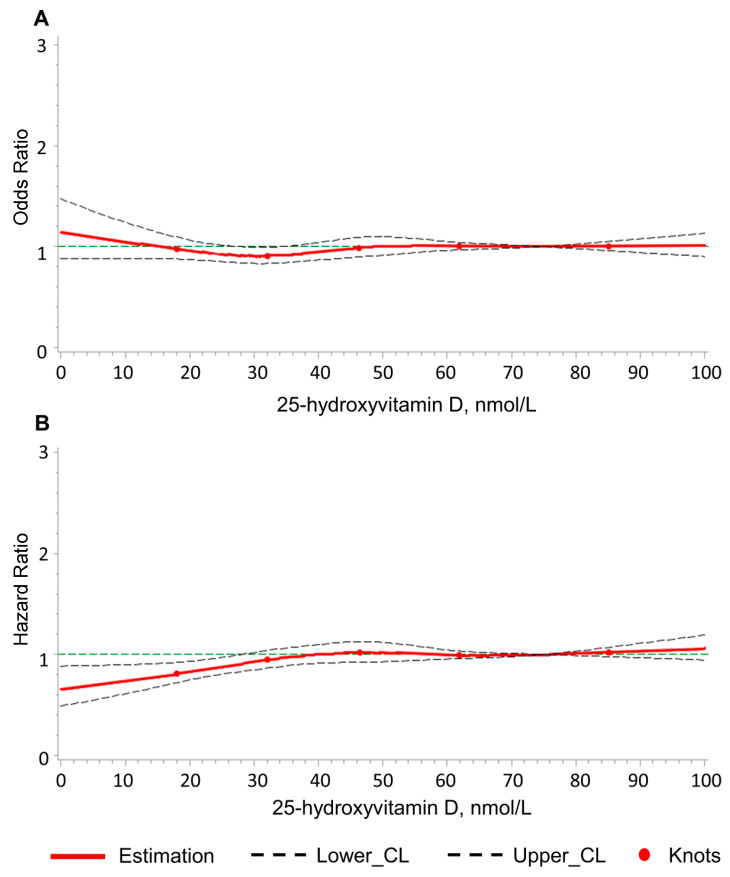
Dose–response relationships of 25-hydroxyvitamin D levels with low back pain, cross-sectionally (**A**) and longitudinally (**B**). Logistic regression (**A**) and Cox proportional hazards regression (**B**) models, adjusted for 27 covariates (see Table S1, Supplemental Materials), using restricted cubic splines with 5 knots positioned at the 5th, 25th, 50th, 75th, and 95th percentiles of the 25(OH)D level. The knots are depicted by dots. The horizontal green lines represent an odds ratio of 1 (**A**) or hazard ratio of 1 (**B**). The solid lines represent the estimated odds ratios or hazard ratios, and the upper and lower dashed lines indicate the upper and lower corresponding 95% confidence intervals.

Figure 2A,B illustrate the dose–response relationship between serum 25(OH)D concentration and LBP in the cross-sectional and longitudinal analyses, respectively. Only the curves for the fully adjusted model are shown. The dose–response analyses exhibited no association cross-sectionally, and a potential reduced risk of LBP longitudinally was observed for 25(OH)D levels in the vitamin D deficiency range below 30 nmol/L. However, the latter finding was likely due to chance, considering the results from multiple testing mentioned above.

### 3.5. Association of Vitamin D Supplements Use and LBP

In comparison to individuals who did not use either vitamin D or multivitamin supplements, those who used vitamin D had a higher likelihood of having LBP after adjusting for age and sex (OR, 95%CI: 1.29, 1.13–1.47) in the cross-sectional analysis (Table 3). This association disappeared after adjusting for all covariates. The longitudinal analyses did not show any associations of vitamin D and multivitamin supplement use with LBP.

Subgroup analyses by age, sex, history of depression and musculoskeletal disease revealed no heterogeneity in the results according to these factors (Table S5, Supplemental Materials).

## 4. Discussion

This study used the data from over 130,000 participants in the large population-based UK Biobank cohort to investigate both the cross-sectional and longitudinal association of 25(OH)D status and vitamin D supplement use with LBP. Age- and sex-adjusted analyses indicated statistically significant associations between vitamin D deficiency and LBP, and between vitamin D supplements use and LBP in cross-sectional analyses. However, both associations disappeared after comprehensive adjustment for potential confounders. In the longitudinal analysis of vitamin D deficiency and LBP, no association with statistical significance was observed in the fully adjusted model. Neither vitamin D insufficiency nor multivitamin use were associated with LBP in any analysis.

The current evidence on the association of 25(OH)D levels with LBP predominately comes from studies with a cross-sectional design. A meta-analysis of 19 cross-sectional studies and 9 case–control studies by Zadro et al. concluded that vitamin D deficiency was associated with LBP [16]. However, most included studies had sample sizes (mostly < 1000 participants) and exhibited high heterogeneity.

A few years after the literature search date for the systematic review in March 2017, a large cross-sectional study involving 17,038 individuals from the Korean National Health and Nutrition Examination Survey was published. This survey observed an inverse association between vitamin D insufficiency and chronic LBP (OR: 0.77, 95% CI: 0.69–0.85) [37] but interpreted it as a lack of an association because there is not biological explanation for such an inverse association. This conclusion also aligns with our findings from the UK Biobank and the sole other prospective study we are aware of. Heuch et al. analyzed the 25(OH)D levels of 1683 incident LBP cases and 3137 controls from a Norwegian community-based cohort in a nested case–control design and found no association (OR per 10 nmol/L 25(OH)D increase: 1.01, 95% CI: 0.97–1.06) [17].

To the best of our knowledge, this study is the first to explore the association of vitamin D supplement use with LBP in a large prospective cohort study. Consistent with the findings for vitamin D status in blood samples, our results for vitamin D supplement use did not observe any association with LBP in fully adjusted models. In line with this observation, a meta-analysis of eight clinical studies by Zadro et al. showed that vitamin D supplementation was not effective in the treatment of LBP when compared with a placebo, no intervention, or other treatments [19].

Although the role of vitamin D in LBP is plausible due to potential anti-inflammatory effects [7] and a general role in maintaining musculoskeletal health [5], the causes of LBP are likely too complex to be significantly influenced by vitamin D supplement use alone. LBP may arise from various factors, such as a sedentary lifestyle, psychosocial issues, injuries, comorbidities, occupational reasons, and genetic predisposition [15]. Future investigations could explore whether vitamin D exerts an impact on distinct etiologies of LBP.

This study has strengths and limitations. The large sample size is a strength, providing high statistical power to detect even weak associations. Furthermore, this is the first cohort study to investigate the longitudinal association of 25(OH)D levels and vitamin D supplement use with LBP, whereas almost all previous studies had a cross-sectional design, which could not differentiate the time sequence of events between vitamin D deficiency/initiation of vitamin D supplement use and LBP. Another strength is obtaining information on vitamin D supplement use from both prescription and OTC medications, as vitamin D supplements are mostly obtained OTC. Furthermore, the question in the interview at baseline about LBP symptoms in the past months ascertains that the study population used for the longitudinal analysis was free of subjects with LBP, allowing for the investigation of true incident cases. In addition, we adjusted for a large number of covariates, thereby minimizing confounding concerns. However, it is important to note that residual confounding cannot be completely disregarded given the nature of observational studies.

A recognized limitation of the UK Biobank is the “healthy volunteer” bias, which primarily influences studies focused on investigating disease prevalence or incidence patterns. However, for our study assessing relative metrics of association, this bias is of no concern. Moreover, the UK Biobank dataset’s partial linkage with primary care records led to the exclusion of approximately 55% of initial cohort participants during the participant selection process. Nonetheless, the distribution of baseline characteristics for the remaining participants in the study was strikingly comparable to that of the complete cohort [26]. A notable limitation is that both the 25(OH)D concentration and the regular use of vitamin D supplement use were ascertained only at the baseline. Potential changes in exposure during follow-up might have hindered observing exposure–outcome associations. However, the proportional hazards assumption was met in all longitudinal analyses, speaking for constant hazards over time and a very limited impact of this limitation on the results. Furthermore, broadly consistent findings between cross-sectional and longitudinal analyses mitigate the impact of this aspect. Finally, it should be noted that this study is primarily generalizable to Caucasians, as the majority of included participants were of this ethnicity.

## 5. Conclusions

Contrary to our initial hypothesis, after comprehensive adjustment for potential confounders, this analysis of over 130,000 participants from the UK Biobank cohort observed neither a statistically significantly increased risk of LBP among subjects with low 25(OH)D levels nor a decreased risk of LBP among vitamin D supplements users. In conclusion, not unexpectedly due to the fact that LBP is multifactorial, our findings provide no evidence for a role of the vitamin D status in the etiology of LBP.

## Figures and Tables

**Table 1:** Baseline Characteristics of Study Population.

Variables	Cross-Sectional Analysis (N = 135,934)	Longitudinal Analysis (N = 130,843)
	N (%)/Median (IQR)	N (%)/Median (IQR)
**Female sex, n (%)**	73,427 (54.0)	70,690 (54.0)
**Age (years), median (IQR)**	58 (50; 63)	58 (50; 63)
**BMI, n (%)**		
<25	46,625 (34.3)	45,351 (34.6)
25–<30	57,440 (42.3)	55,317 (42.3)
≥30	31,368 (23.1)	29,703 (22.7)
**Smoking, n (%)**		
Never	76,907 (56.6)	74,471 (56.9)
Ever	58,990 (43.4)	56,337 (43.1)
**Hypertension, n (%)**	35,014 (25.8)	33,359 (25.5)
**Diabetes, n (%)**	6286 (4.6)	5957 (4.6)
**CHD, n (%)**	5797 (4.3)	5429 (4.2)
**Lifetime history of depression, n (%)**	14,614 (10.8)	13,842 (10.6)
**No. of chronic diseases, median (IQR)**	1 (0; 3)	1 (0; 3)
**Lifetime history of musculoskeletal diseases, n (%)**	72,784 (53.5)	68,524 (52.4)
**Lifetime history of injury to the abdomen, lower back, lumbar spine and pelvis, n (%)**	2674 (2.0)	2384 (1.8)
**Low back pain in the month before enrolment, n (%)**	5091 (3.8)	N.A.
**Low back pain during follow-up, n (%)**	N.A.	4288 (3.3)
**25(OH)D concentration (nmol/L), median (IQR)**	46.3 (32; 61.9)	46.4 (32; 61.9)
**Vitamin D status, n (%)**		
Deficiency (<30 nmol/L)	29,419 (21.6)	28,216 (21.6)
Insufficiency (30–<50 nmol/L)	46,949 (34.5)	45,208 (34.6)
Sufficiency (≥50 nmol/L)	59,566 (43.8)	57,419 (43.9)
**Vitamin D intake, n (%)**		
No	103,710 (76.3)	99,886 (76.3)
Multivitamins ± minerals	26,807 (19.7)	25,792 (19.7)
Vitamin D	5417 (4.0)	5165 (3.8)

Note: BMI: body mass index, CHD: coronary heart disease, 25(OH)D: 25-hydroxy-vitamin D, IQR: interquartile range, N.A.: Not applicable.

**Table 2:** Associations of vitamin D deficiency and insufficiency with low back pain, cross-sectionally and longitudinally.

	Vitamin D Status									
	Deficiency				Insufficiency				Sufficiency	
	N_case_ (%)	OR/HR (95% CI)	*p*-Value ^a^		N_case_ (%)	OR/HR (95% CI)	*p*-Value ^a^		N_case_ (%)	OR/HR (95% CI)
**Cross-sectional analyses**										
Adjusted for age & sex	1203 (4.1)	1.13 (1.05, 1.22)	0.0008		1741 (3.7)	1.03 (0.96, 1.10)	0.42		2147 (3.6)	Ref
Adjusted for all covariates ^b^	1203 (4.1)	0.95 (0.87, 1.03)	0.21		1741 (3.7)	0.97 (0.91, 1.04)	0.38		2147 (3.6)	Ref
**Longitudinal analyses**										
Adjusted for age & sex	874 (3.1)	0.93 (0.86, 1.01)	0.07		1533 (3.4)	1.03 (0.96, 1.10)	0.45		1881 (3.3)	Ref
Adjusted for all covariates ^b^	874 (3.1)	0.87 (0.79, 0.95)	0.0032		1533 (3.4)	1.00 (0.93, 1.07)	0.96		1881 (3.3)	Ref

Abbreviation: CI: confidence interval, HR: hazard ratio, OR: odds ratio, Ref: reference. ^a^ A *p*-value < 0.003125 indicates statistical significance after correction for multiple testing for 16 tests by the Bonferroni method. ^b^ Model adjusted for n = 27 variables listed in Table S1 (Supplemental Materials).

**Table 3:** Associations of vitamin D and multivitamin supplements use with low back pain, cross-sectionally and longitudinally.

	Vitamin Use									
	Non-Users			Multivitamin Users				Vitamin D Users		
	N_case_ (%)	OR/HR (95%CI)		N_case_ (%)	OR/HR (95%CI)	*p*-Value ^a^		N_case_ (%)	OR/HR (95%CI)	*p*-Value ^a^
**Cross-sectional analyses**										
Adjusted for age & sex	3824 (3.7)	Ref		1015 (3.8)	1.03 (0.96, 1.11)	0.42		252 (4.7)	1.29 (1.13, 1.47)	0.0001
Adjusted for all covariates ^b^	3824 (3.7)	Ref		1015 (3.8)	0.97 (0.90, 1.05)	0.45		252 (4.7)	0.99 (0.86, 1.14)	0.87
**Longitudinal analyses**										
Adjusted for age & sex	3269 (3.3)	Ref		853 (3.3)	1.01 (0.94, 1.09)	0.69		166 (3.2)	0.99 (0.85, 1.16)	0.90
Adjusted for all covariates ^b^	3269 (3.3)	Ref		853 (3.3)	0.99 (0.91, 1.07)	0.74		166 (3.2)	0.93 (0.80, 1.09)	0.38

Abbreviation: CI: confidence interval, HR: hazard ratio, OR: odds ratio, Ref: reference. ^a^ A *p*-value < 0.003125 indicates statistical significance after correction for multiple testing for 16 tests by the Bonferroni method. ^b^ Model adjusted for n = 30 variables listed in Table S1 (Supplemental Materials).

## Data Availability

This study utilized data from the UK Biobank. Access to publicly available data is provided to researchers through an open application process at https://www.ukbiobank.ac.uk/register-apply/, accessed on 20 September 2023.

## Supplementary Materials

The following supporting information can be downloaded at: https://www.mdpi.com/article/10.3390/nu16060806/s1, Table S1. List of baseline characteristics adjusted for in the analyses on vitamin D status and vitamin D supplementation. Table S2. Distribution of full list of baseline characteristics of the study population in the cross-sectional and longitudinal analyses. Table S3. Cross-sectional association of covariates with low back pain. Table S4. Subgroup analyses on the associations of vitamin D deficiency and insufficiency with low back pain, cross-sectionally and longitudinally. Table S5. Subgroup analyses on the associations of vitamin D supplement and multivitamin use with low back pain, cross-sectionally and longitudinally.

## Abbreviations

25(OH)D	25-hydroxyvitamin D
BMI	body mass index
CHD	coronary heart disease
CI	confidence interval
EMIS	Egton Medical Information Systems
GP	general practitioner
HR	hazard ratio
ICD-10	International Statistical Classification of Diseases
IQR	interquartile range
LBP	low back pain
N.A.	not applicable
NHS	National Health Service
OTC	over-the-counter
RCS	restricted cubic splines
Ref	reference
RIQAS	Randox International Quality Assessment Scheme
SAS	Statistical Analysis System
UK	United Kingdom
VIF	variation inflation factor
