# Supplementary material for: Serum 25-Hydroxyvitamin D Status and Vitamin D Supplements Use Are Not Associated with Low Back Pain in the Large UK Biobank Cohort
Source: Nutrients. 2024 Mar 12;16(6):806. doi: 10.3390/nu16060806 (PMC10974643; doi:10.3390/nu16060806)
Supplement: Supplementary file 1 [file nutrients-16-00806-s001.zip › nutrients-2887342-supplementary.pdf]

Supplemental Materials

Table of Contents

Table S1. List of baseline characteristics adjusted for in the analyses on vitamin D status and vitamin D supplementation ... 2

Table S2. Distribution of full list of baseline characteristics of the study population in the cross-sectional and longitudinal analyses ... 3

Table S3. Cross-sectional association of covariates with low back pain at baseline ... 6

Table S4. Subgroup analyses on the associations of vitamin D deficiency and insufficiency with low back pain, cross-sectionally and longitudinally ... 9

Table S5. Subgroup analyses on the associations of vitamin D supplement and multivitamin use with low back pain, cross-sectionally and longitudinally ... 10

Table S1. List of baseline characteristics adjusted for in the analyses on vitamin D status and vitamin D supplementation.

Variables	Covariate in the Analyses on Vitamin D Status (n = 27)	Covariate in the Analyses on Vitamin D Supplementation (n = 30)
**SOCIO-DEMOGRAPHIC/-ECONOMIC FACTORS**		
Age	Yes	Yes
Sex	Yes	Yes
Education	Yes	No
Annual household income	Yes	Yes
**LIFE-STYLE FACTORS**		
Smoking	Yes	Yes
Venturesome personality	No	Yes
Total physical activity	Yes	Yes
**DISEASES & DISEASE SYMPTOMS**		
Diabetes	Yes	Yes
Stroke	Yes	Yes
Coronary heart disease	Yes	Yes
Hypertension	No	Yes
Tiredness/lethargy in last 2 weeks	Yes	Yes
Depressed mood	Yes	Yes
History of depression	No	Yes
History of musculoskeletal disease	Yes	Yes
History of injury to abdomen, lower back, lumbar spine and pelvis	Yes	Yes
Cancer	No	Yes
**BIOMARKERS**		
Body mass index	Yes	Yes
Systolic blood pressure	Yes	No
Forced expiratory volume in 1-second	Yes	No
Hand grip strength	Yes	No
**GENERAL HEALTH**		
Disability	Yes	Yes
General self-reported health	Yes	Yes
No of chronic diseases	Yes	Yes
No of drugs	Yes	Yes
Low-dose aspirin use	No	Yes
Lipid-lowering drugs use	No	Yes
Anti-depressants use	No	Yes
**VITAMIN D SPECIFIC FACTORS**		
Latitude of study center	Yes	Yes
Month of attending the study center	Yes	Yes
Time spent outdoors in summer	Yes	Yes
Skin color brown/black	Yes	Yes
Ease of skin tanning	Yes	Yes
Solarium/sunlamp use	Yes	Yes

Table S2. Distribution of full list of baseline characteristics of the study population in the cross-sectional and longitudinal analyses.

Variables	Cross-Sectional Analysis N(%)[b]/Median (IQR)	Longitudinal Analysis [a] N(%)[b]/Median (IQR)
N_{total}	135,934	130,843
**SOCIO-DEMOGRAPHIC/-ECONOMIC FACTORS**		
**Age (years), median (IQR)**	58 (50; 63)	58 (50; 63)
**Sex, n (%)**		
Female	73,427 (54.0)	70,690 (54.0)
Male	62,507 (46.0)	60,153 (46.0)
**Education (years), median (IQR)**	12 (10; 17)	12 (10; 17)
**Annual household income (£), n (%)**		
< 18,000	25,666 (18.9)	24,310 (18.6)
18,000 - < 51,999	61,611 (45.3)	59,470 (45.5)
52,000 - < 100,000	24,065 (17.7)	23,431 (17.9)
≥ 100,000	6269 (4.6)	6141 (4.7)
**LIFESTYLE FACTORS**		
**Smoking, n (%)**		
Never	76,907 (56.6)	74,471 (56.9)
Ever	58,990 (43.4)	56,337 (43.1)
**Venturesome personality, n (%)**		
No	96,988 (71.4)	93,542 (71.5)
Yes	34,087 (25.1)	32,644 (25.0)
**Total physical activity (hours/day), n (%)**		
≤ 1	20,158 (14.8)	19,212 (14.7)
≤ 2	45,388 (33.4)	43,979 (33.6)
> 2	45,390 (33.4)	43,839 (33.5)
**DISEASES & DISEASE SYMPTOMS**		
**Diabetes, n (%)**		
No	129,609 (95.4)	124,850 (95.4)
Yes	6286 (4.6)	5957 (4.6)
**Stroke, n (%)**		
No	134,198 (98.7)	129,207 (98.8)
Yes	1696 (1.3)	1599 (1.2)
**Coronary heart disease, n (%)**		
No	130,097 (95.7)	125,377 (95.8)
Yes	5797 (4.3)	5429 (4.2)
**Hypertension, n (%)**		
No	100,883 (74.2)	97,450 (74.5)
Yes	35,014 (25.7)	33,359 (25.5)
**History of depression, n (%)**		
No	121,281 (89.2)	116,965 (89.4)
Yes	14,614 (10.8)	13,842 (10.6)
**Frequency of depressed mood in last 2 weeks, n (%)**		
≤ half the days	124,873 (91.9)	120,528 (92.1)

Variables	Cross-Sectional Analysis N(%)[b]/Median (IQR)	Longitudinal Analysis [a] N(%)[b]/Median (IQR)
**N_{total}**	135,934	130,843
> half the days	5448 (4.0)	5004 (3.8)
**Tiredness/lethargy in last 2 weeks, n (%)**		
≤ half the days	117,967 (86.8)	114,150 (87.2)
> half the days	13,967 (10.3)	12,860 (9.8)
**History of musculoskeletal disease, n (%)**		
No	63,150 (46.5)	62,319 (47.6)
Yes	72,784 (53.5)	68,524 (52.4)
**History of injury to abdomen, lower back, lumbar spine and pelvis, n (%)**		
No	133,260 (98.0)	128,459 (98.2)
Yes	2674 (2.0)	2384 (1.8)
**Cancer, n (%)**		
No	125,650 (92.4)	120,951 (92.4)
Yes	9922 (7.3)	9548 (7.3)
BIOMARKERS		
**Body mass index (kg/m²), n (%)**		
Underweight, < 18.5	709 (0.5)	685 (0.5)
Normal weight, 18.5 - <25	45,916 (33.8)	44,666 (34.1)
Overweight: 25 - < 30	57,440 (42.3)	55,317 (42.3)
Obesity class: ≥ 30	31,368 (23.1)	29,703 (22.7)
**Systolic blood pressure (mmHg), n (%)**		
< 140	70,852 (52.1)	68,045 (52.0)
140 - < 160	43,070 (31.7)	41,497 (31.7)
160 - < 180	17,257 (12.7)	16,698 (12.8)
≥ 180	4590 (3.4)	4448 (3.4)
**Forced expiratory volume in 1-second (L), median (IQR)**	2.8 (2.3; 3.3)	2.8 (2.3; 3.3)
**Hand grip strength (Kg), median (IQR)**	31 (24; 41)	31 (24; 41)
GENERAL HEALTH		
**Disability (%)**		
No	128,905 (94.8)	124,728 (95.3)
Yes	6002 (4.4)	5165 (4.0)
**General self-reported health, n (%)**		
Excellent	25,577 (18.8)	25,248 (19.3)
Good	80,962 (59.6)	78,609 (60.1)
Fair	24,415 (18.0)	22,642 (17.3)
Poor	4472 (3.3)	3867 (3.0)
**No of chronic diseases, median (IQR)**	1 (0; 3)	1 (0; 3)
**No of drugs, median (IQR)**	2 (0; 3)	2 (0; 3)
**Low-dose aspirin use, n (%)**		
No	117,001 (86.1)	112,774 (86.2)
Yes	18,893 (13.9)	18,032 (13.8)
**Lipid-lowering drugs use, n (%)**		
No	112,724 (82.9)	108,794 (83.2)

Variables	Cross-Sectional Analysis N(%)[b]/Median (IQR)	Longitudinal Analysis [a] N(%)[b]/Median (IQR)
**N_{total}**	135,934	130,843
Yes	23,172 (17.1)	22,014 (16.8)
**Anti-depressants use, n (%)**		
No	128,132 (94.3)	123,729 (94.6)
Yes	7762 (5.7)	7077 (5.4)
**VITAMIN D SPECIFIC FACTORS**		
**Latitude of study center (per 1°), median (IQR)**	53.4 (52.5; 54.6)	53.4 (52.5; 54.6)
**Calendar month of attending the study center**		
1	10,791 (7.9)	10,358 (7.9)
2-3	24,925 (18.3)	23,917 (18.3)
4	12,267 (9.0)	11,725 (9.0)
5	13,516 (9.9)	12,936 (9.9)
6	12,499 (9.2)	12,074 (9.2)
7	10,338 (7.6)	9977 (7.6)
8	10,705 (7.9)	10,362 (7.9)
9	9600 (7.1)	9294 (7.1)
10	11,331 (8.3)	10,959 (8.4)
11	12,396 (9.1)	11,951 (9.1)
12	7566 (5.6)	7290 (5.6)
**Time spent outdoors in summer (h/day), n (%)**		
<1	5506 (4.1)	5281 (4.0)
1-2	40,223 (29.6)	39,019 (29.8)
3-4	42,876 (31.5)	41,249 (31.5)
5-6	25,682 (18.9)	24,675 (18.9)
≥ 7	14,310 (10.5)	13,621 (10.4)
**Skin color, n (%)**		
Light (fair - olive)	127800 (94.0)	123,111 (94.1)
Brown	2428 (1.8)	2319 (1.8)
Black	3167 (2.3)	3006 (2.3)
**Ease of skin tanning, n (%)**		
Very tanned	27,287 (20.1)	26,106 (20.0)
Moderately tanned	53,107 (39.1)	51,233 (39.2)
Mildly/occasionally tanned	28,599 (21.0)	27,589 (21.1)
Never tan, only burn	23,537 (17.3)	22,653 (17.3)
**Solarium/sunlamp use (times per year), n (%)**		
Never	121,854 (89.6)	117,433 (89.8)
< 1	6652 (4.9)	6385 (4.9)
1 - 6	3316 (2.4)	3143 (2.4)
7 - 12	1450 (1.1)	1374 (11)
> 12	1386 (1.0)	1308 (1.0)

Abbreviations: IQR: interquartile range.

[a] Population with low back pain before/at baseline not included.

[b] Denominators in proportion calculations contain missing values

Table S3. Cross-sectional association of covariates with low back pain at baseline.

Variable	OR (95%CI)
**SOCIO-DEMOGRAPHIC/ -ECONOMIC FACTORS**	
**Age (years), median (IQR)**	0.98 (0.98, 0.99)
**Sex, n (%)**	
Female	Ref
Male	0.75 (0.68, 0.83)
**Education (years), median (IQR)**	0.96 (0.95, 0.97)
**Annual household income (£), n (%)**	
< 18,000	Ref
18,000 - < 51,999	0.93 (0.86, 1.00)
52,000 - < 100,000	0.85 (0.76, 0.96)
≥ 100,000	0.78 (0.63, 0.96)
**LIFESTYLE FACTORS**	
**Smoking, n (%)**	
Never	Ref
Ever	1.34 (1.22, 1.47)
**Venturesome personality, n (%)**	
No	Ref
Yes	1.12 (1.05, 1.20)
**Total physical activity (hours/day), n (%)**	
≤ 1	Ref
≤ 2	0.88 (0.81, 0.96)
> 2	0.96 (0.87, 1.06)
**DISEASES & DISEASE SYMPTOMS**	
**Diabetes, n (%)**	
No	Ref
Yes	0.80 (0.75, 0.85)
**Stroke, n (%)**	
No	Ref
Yes	0.64 (0.51, 0.81)
**Coronary heart disease, n (%)**	
No	Ref
Yes	0.81 (0.71, 0.93)
**Hypertension, n (%)**	
No	Ref
Yes	0.80 (0.74, 0.87)
**History of depression, n (%)**	
No	Ref
Yes	0.81 (0.74, 0.89)
**Frequency of depressed mood in last 2 weeks, n (%)**	
≤ half the days	Ref
> half the days	1.16 (1.03, 1.31)

Variable	OR (95%CI)
**Tiredness/lethargy in last 2 weeks, n (%)**	
≤ half the days	Ref
> half the days	1.23 (1.13, 1.34)
**History of musculoskeletal disease, n (%)**	
No	Ref
Yes	3.79 (3.51, 4.09)
**History of injury to abdomen, lower back, lumbar spine and pelvis, n (%)**	
No	Ref
Yes	2.24 (1.96, 2.56)
**Cancer, n (%)**	
No	Ref
Yes	0.75 (0.67, 0.84)
BIOMARKERS	
**Body mass index (kg/m^2), n (%)**	
Underweight, < 18.5	0.95 (0.62, 1.46)
Normal weight, 18.5 - < 25	Ref
Overweight: 25 - < 30	1.13 (1.05, 1.22)
Obesity class: ≥ 30	1.13 (1.04, 1.23)
**Systolic blood pressure (mmHg), n (%)**	
< 140	Ref
140 - < 160	0.89 (0.83, 0.96)
160 - < 180	0.80 (0.72, 0.88)
≥ 180	0.81 (0.68, 0.97)
**Forced expiratory volume in 1-second (L), median (IQR)**	1.07 (1.02, 1.13)
**Hand grip strength (Kg), median (IQR)**	1.20 (1.14, 1.25)
GENERAL HEALTH	
**Disability (%)**	
No	Ref
Yes	1.76 (1.59, 1.95)
**General self-reported health, n (%)**	
Excellent	Ref
Good	1.85 (1.65, 2.09)
Fair	3.36 (2.96, 3.82)
Poor	3.53 (2.98, 4.19)
**No of chronic diseases, median (IQR)**	1.11 (1.09, 1.13)
**No of drugs, median (IQR)**	1.10 (1.09, 1.12)
**Low-dose aspirin use, n (%)**	
No	Ref
Yes	0.88 (0.80, 0.97)
**Lipid-lowering drugs use, n (%)**	

Variable	OR (95%CI)
No	Ref
Yes	0.86 (0.78, 0.94)
**Anti-depressants use, n (%)**	
No	Ref
Yes	1.17 (1.06, 1.30)
**VITAMIN D SPECIFIC FACTORS**	
**Latitude of study center (per 1°), median**	0.88 (0.86, 0.90)
**Calendar month of attending the study center**	
1	Ref
2-3	0.98 (0.87, 1.10)
4	1.09 (0.95, 1.24)
5	1.02 (0.90, 1.17)
6	0.82 (0.71, 0.94)
7	0.82 (0.71, 0.95)
8	0.77 (0.67, 0.90)
9	0.79 (0.68, 0.92)
10	0.82 (0.71, 0.95)
11	0.93 (0.81, 1.07)
12	0.94 (0.80, 1.10)
**Time spent outdoors in summer (h/day), n (%)**	
<1	Ref
1-2	0.95 (0.82, 1.11)
3-4	1.13 (0.96, 1.32)
5-6	1.13 (0.96, 1.32)
≥ 7	1.30 (1.10, 1.54)
**Skin color, n (%)**	
Light (fair - olive)	Ref
Brown	1.23 (1.03, 1.47)
Black	1.15 (0.80, 1.66)
**Ease of skin tanning, n (%)**	
Very tanned	Ref
Moderately tanned	0.94 (0.87, 1.01)
Mildly/occasionally tanned	0.93 (0.85, 1.02)
Never tan, only burn	0.91 (0.83, 1.00)
**Solarium/sunlamp use (times per year), n (%)**	
Never	Ref
< 1	1.10 (0.97, 1.26)
1 - 6	1.35 (1.14, 1.59)
7 - 12	1.37 (1.07, 1.75)
> 12	1.39 (1.09, 1.77)

Abbreviation: CI: confidence interval, OR: odds ratio, Ref: reference.

Table S4. Subgroup analyses on the associations of vitamin D deficiency and insufficiency with low back pain, cross-sectionally and longitudinally.

Study population	Cross-sectional analyses						Longitudinal analyses					
	Vitamin D status						Vitamin D status					
	Deficiency		Insufficiency		Sufficiency		Deficiency		Insufficiency		Sufficiency	
	N_{case} (%)	OR (95%CI)	N_{case} (%)	OR (95%CI)	N_{case} (%)	OR (95%CI)	N_{case}(%)	HR (95%CI)	N_{case} (%)	HR (95%CI)	N_{case} (%)	HR (95%CI)
**By age**												
<65 years	1039 (4.1)	0.94 (0.86, 1.03)	1452 (3.8)	0.98 (0.90, 1.05)	1722 (3.7)	Ref	755 (3.1)	0.87 (0.79, 0.97) **	1255 (3.4)	1.00 (0.92, 1.08)	1481 (3.3)	Ref
≥65 years	164 (3.9)	1.00 (0.81, 1.23)	289 (3.4)	0.95 (0.81, 1.12)	425 (3.4)	Ref	119 (3.0)	0.85 (0.68, 1.07)	278 (3.4)	0.99 (0.85, 1.17)	400 (3.3)	Ref
**By sex**												
Females	657 (4.1)	0.97 (0.87, 1.09)	925 (3.7)	0.97 (0.88, 1.07)	1155 (3.6)	Ref	487 (3.2)	0.93 (0.82, 1.05)	841 (3.5)	1.05 (0.96, 1.16)	1007 (3.2)	Ref
Males	546 (4.0)	0.93 (0.81, 1.05)	816 (3.8)	0.97 (0.88, 1.08)	992 (3.65)	Ref	387 (3.0)	0.82 (0.71, 0.94)	692 (3.3)	0.94 (0.85, 1.05)	874 (3.3)	Ref
**By history of depression**												
Yes	179 (5.6)	0.76 (0.61,0.96) *	270 (5.4)	0.91 (0.75, 1.09)	324 (5.0)	Ref	108 (3.6)	0.85 (0.65, 1.11)	166 (3.5)	0.90 (0.73, 1.11)	233 (3.8)	Ref
No	1024 (3.9)	0.98 (0.90, 1.08)	1471 (3.5)	0.98 (0.91, 1.06)	1823 (3.4)	Ref	766 (3.0)	0.88 (0.79, 0.97) *	1367 (3.4)	1.01 (0.94, 1.09)	1648 (3.2)	Ref
**By history of musculoskeletal disease**												
Yes	994 (6.9)	0.97 (0.88, 1.07)	1455 (5.9)	0.99 (0.91, 1.06)	1811 (5.4)	Ref	532 (4.0)	0.90 (0.80, 1.01)	971 (4.2)	1.01 (0.92, 1.10)	1242 (3.9)	Ref
No	209 (1.4)	0.84 (0.69, 1.03)	286 (1.3)	0.90 (0.76, 1.07)	336 (1.3)	Ref	342 (2.3)	0.82 (0.70, 0.95) *	562 (2.6)	0.98 (0.87, 1.10)	639 (2.5)	Ref

Abbreviation: CI: confidence interval, HR: hazard ratio, OR: odds ratio, Ref: reference.

* p<0.05, ** p<0.001. No results were statistically significant after correction for multiple testing with Bonferroni's method for the n=64 tests conducted for the subgroup analyses shown in Suppl. Table S4 and S5.

[a] Subgroup analyses adjusted for all covariates listed in **Table S1 (Suppl. Material)**, except for the one used for categorizing subgroups.

Table S5. Subgroup analyses on the associations of vitamin D supplement and multivitamin use with low back pain, cross-sectionally and longitudinally.

Study population	Cross-sectional analyses						Longitudinal analyses					
	Vitamin supplement use						Vitamin supplement use					
	Non-users		Multivitamin		Vitamin D		Non-users		Multivitamin		Vitamin D	
	N_{case} (%)	OR[a] (95%CI)	N_{case} (%)	OR[a] (95%CI)	N_{case} (%)	OR (95%CI)	N_{case} (%)	HR[a] (95%CI)	N_{case} (%)	HR[a] (95%CI)	N_{case} (%)	HR[a] (95%CI)
**By age**												
<65 years	3173 (3.8)	Ref	848 (3.8)	0.96 (0.88, 1.04)	192 (4.6)	0.96 (0.82, 1.13)	2658 (3.3)	Ref	706 (3.3)	0.99 (0.91, 1.08)	127 (3.2)	0.95 (0.79, 1.14)
≥65 years	651 (3.4)	Ref	167 (3.6)	1.06 (0.88, 1.27)	60 (4.8)	1.20 (0.90, 1.60)	611 (3.3)	Ref	147 (3.3)	0.99 (0.82, 1.19)	39 (3.3)	0.95 (0.68, 1.32)
**By sex**												
Females	1969 (3.7)	Ref	597 (3.7)	0.93 (0.84, 1.03)	171 (4.45)	0.96 (0.81, 1.14)	1704 (3.3)	Ref	515 (3.3)	0.96 (0.87, 1.06)	116 (3.2)	0.92 (0.76, 1.11)
Males	1855 (3.7)	Ref	418 (4.0)	1.04 (0.93, 1.17)	81 (5.1)	1.11 (0.87, 1.41)	1565 (3.2)	Ref	338 (3.4)	1.04 (0.92, 1.17)	50 (3.3)	1.01 (0.76, 1.34)
**By history of depression**												
Yes	575 (5.4)	Ref	159 (4.9)	0.94 (0.78, 1.14)	39 (5.7)	0.84 (0.58, 1.20)	367 (3.6)	Ref	109 (3.5)	0.97 (0.78, 1.21)	31 (4.8)	1.25 (0.86, 1.83)
No	3249 (3.5)	Ref	856 (3.6)	0.99 (0.91, 1.07)	213 (4.5)	1.05 (0.90, 1.22)	2902 (3.2)	Ref	744 (3.3)	0.99 (0.91, 1.08)	135 (3.0)	0.89 (0.75, 1.07)
**By history of musculoskeletal disease**												
Yes	3173 (5.8)	Ref	863 (5.9)	1.01 (0.93, 1.10)	224 (6.8)	1.05 (0.90, 1.22)	2090 (4.0)	Ref	537 (3.9)	0.96 (0.88, 1.06)	118 (3.8)	0.96 (0.79, 1.15)
No	651 (1.3)	Ref	152 (1.2)	0.84 (0.70, 1.00)	28 (1.3)	0.82 (0.56, 1.22)	1179 (2.5)	Ref	316 (2.6)	1.04 (0.92, 1.19)	48 (2.3)	0.93 (0.69, 1.25)

Abbreviation: CI: confidence interval, HR: hazard ratio, OR: odds ratio, Ref: reference.

[a] Subgroup analyses adjusted for all covariates listed in **Table S1 (Suppl. Material)**, except for the one used for categorizing subgroups.
